# Pallidal Deep Brain Stimulation for Monogenic Dystonia: The Effect of Gene on Outcome

**DOI:** 10.3389/fneur.2020.630391

**Published:** 2021-01-08

**Authors:** Stephen Tisch, Kishore Raj Kumar

**Affiliations:** ^1^Department of Neurology, St Vincent's Hospital, University of New South Wales, Sydney, NSW, Australia; ^2^Molecular Medicine Laboratory and Neurology Department, Concord Clinical School, Concord Repatriation General Hospital, The University of Sydney, Sydney, NSW, Australia; ^3^Kinghorn Centre for Clinical Genomics, Garvan Institute of Medical Research, Darlinghurst, NSW, Australia

**Keywords:** dystonia, deep brain stimulation, globus pallidus internus, pallidal, gene, mutation, monogenic

## Abstract

Globus pallidus internus deep brain stimulation (GPi DBS) is the most effective intervention for medically refractory segmental and generalized dystonia in both children and adults. Predictive factors for the degree of improvement after GPi DBS include shorter disease duration and dystonia subtype with idiopathic isolated dystonia usually responding better than acquired combined dystonias. Other factors contributing to variability in outcome may include body distribution, pattern of dystonia and DBS related factors such as lead placement and stimulation parameters. The responsiveness to DBS appears to vary between different monogenic forms of dystonia, with some improving more than others. The first observation in this regard was reports of superior DBS outcomes in DYT-TOR1A (DYT1) dystonia, although other studies have found no difference. Recently a subgroup with young onset DYT-TOR1A, more rapid progression and secondary worsening after effective GPi DBS, has been described. Myoclonus dystonia due to DYT-SCGE (DYT11) usually responds well to GPi DBS. Good outcomes following GPi DBS have also been documented in X-linked dystonia Parkinsonism (DYT3). In contrast, poorer, more variable DBS outcomes have been reported in DYT-THAP1 (DYT6) including a recent larger series. The outcome of GPi DBS in other monogenic isolated and combined dystonias including DYT-GNAL (DYT25), DYT-KMT2B (DYT28), DYT-ATP1A3 (DYT12), and DYT-ANO3 (DYT24) have been reported with varying results in smaller numbers of patients. In this article the available evidence for long term GPi DBS outcome between different genetic dystonias is reviewed to reappraise popular perceptions of expected outcomes and revisit whether genetic diagnosis may assist in predicting DBS outcome.

## Introduction

Dystonia is a chronic neurological condition characterized by sustained or intermittent muscle contractions resulting in abnormal movements, postures and tremor (1, 2). Genetic dystonias can be defined as those in which an underlying gene is identified as the cause. The genetic understanding of dystonia has expanded with an increasing number of dystonia genes identified. The identification of causative genes has reduced the number of truly idiopathic cases, as many patients previously classified as having idiopathic isolated dystonia can now be attributed to a specific gene. Some genetic dystonias have neurological features beyond pure dystonia and are classified as combined phenotypes. Conceptual shifts in dystonia classification, improved genetic diagnosis, and recognition of phenotypic spectrums for individual genes have allowed for better characterization of dystonic syndromes and their response to treatment both medical and surgical. Globus pallidus internus (GPi) deep brain stimulation (DBS) is the most effective known treatment for medically refractory dystonia with established efficacy in segmental and generalized dystonia of idiopathic, genetic or acquired causes. The potency and versatility of GPi DBS has encouraged its use in an expanding range of medically refractory genetic dystonias with varying degrees of success. In this article we review available information for the effectiveness of GPi DBS in dystonia due to various causative genes in an effort to stratify whether genetic diagnosis may offer some assistance in predicting the DBS treatment outcome.

## Overview of GPi DBS in Dystonia

GPi-DBS is effective in medically refractory dystonia (3–8). Improvement in dystonia after GPi DBS is progressive over months (5, 9–11). Mobile components of dystonia tend to respond more quickly than the tonic elements (12, 13). Isolated dystonia either idiopathic or genetic (primary) dystonia improves to a greater degree than acquired combined (secondary) dystonia (14–17). However, exceptions include acquired tardive dystonia secondary to neuroleptic exposure, (18) and dystonia in neurodegeneration with brain iron accumulation (19, 20), which respond well to GPi DBS (see section on NBIA/DYT-*PANK2*). Conversely idiopathic isolated craniofacial and laryngeal dystonia may show a poorer than expected response to DBS (21), emphasizing the importance of not only etiology but also body distribution on DBS dystonia outcome. Shorter disease duration has also been correlated with improved GPi DBS outcome (22). Technical aspects of GPi DBS including electrode placement and stimulation parameters also have a significant effect on DBS outcome. Chronic stimulation in the most posteroventral portion of GPi is most effective (23). Dystonia DBS typically requires higher electrical parameters than STN DBS for Parkinson's disease, however longer stimulation pulse widths above 60 ms are not beneficial and are less energy efficient (24–26), while lower frequency stimulation below 100 Hz may be useful in selected patients (27). Rare treatment failures after GPi DBS may occur in all categories of dystonia including genetic isolated generalized dystonia and appear independent of technical reasons such poor lead placement (28).

## Genetic Forms of Dystonia and Response to GPi-DBS

We review the evidence for GPi-DBS in individual genetic forms of dystonia (Supplementary Table 1).

### DYT-*TOR1A* (DYT1)

DYT-*TOR1A* (DYT1) is the most common cause of young onset familial isolated dystonia and typically begins between 9 and 12 years in a limb then spreads to become generalized with relative sparing of cervical and bulbar segments (29, 30). Among the earliest reports of GPi DBS for generalized dystonia was the study of Coubes et al. reporting 6 children and 1 adult with medically refractory DYT1 generalized dystonia with remarkable improvement after bilateral GPi DBS, resulting in a mean improvement in Burke-Fahn-Marsden Dystonia rating scale (BFMDS) motor score of 90% at 12 months (3). The same group reported a tendency for superior GPi DBS outcome in DYT1 dystonia in 15 patients compared with 17 idiopathic non-DYT1 patients (31). Krause et al. reported superior GPi DBS outcome in 4 DYT1 dystonia patients compared with 6 non-DYT1 patients, with improvements in BFMDS of 55.6 and 35.1% respectively, although one DYT1 patient showed secondary worsening 3 years after DBS (12). Starr et al. also reported marked but variable benefit in DYT1 dystonia following GPi DBS with improvement approaching 100% in two patients, 51% in another and 14% in a patient with long disease duration and fixed orthopedic deformity, factors known to limit overall benefit (32, 33). Longer term studies of GPi DBS in DYT1 have shown sustained benefit for up to 10 years (34) including a large study of 47 DYT1 patients treated with GPi DBS which reported average long-term improvement approaching 80% (35). Some studies have reported a statistically superior outcome of GPi DBS in DYT1 dystonia compared with non-DYT1 dystonia (23), however larger studies with rater-blinded methodology have shown no significant difference (5, 6). A meta regression study by Andrews et al. reviewed individual patient outcomes of GPi DBS in 466 patients including 91 DYT1 and 108 non-DYT generalized dystonia patients. They found DYT1 patients improved by 67.5% in BFMDS compared with 55.8% for non-DYT, and in multivariate analysis DYT1 was in independent predictor of superior outcome, along with shorter disease duration and lower baseline severity score (16). A subsequent larger meta regression study identified DYT1 as a predictor of improved outcome in univariate analysis but found only higher baseline severity score as a significant predictor of superior outcome in multivariate analysis, however, DYT1 status was excluded from the multivariate analysis owing to some studies not reporting DYT1 status (36). A systematic review of GPi DBS outcomes in children found a higher probability of >50% improvement in DYT1 vs. non-DYT1- patients (65 vs 29%) and higher percentage improvement among DYT1 patients compared with non-DYT1 (66 vs. 43%) but not reaching statistical significance (37).

While DYT1 dystonia usually responds well to GPi DBS, especially in patients with shorter disease duration and without orthopedic deformity, there are important exceptions where secondary worsening of dystonia may occur. Cif et al. described secondary worsening after several years in a subgroup of DYT1 dystonia patients with good initial response at 12 months (34). A recent multicentre study reported secondary worsening in DYT1 dystonia after GPi DBS in 11 of 132 patients at 6 months to 3 years after DBS and was associated with younger age of onset, faster disease progression and cranial involvement (38).

### DYT-*THAP1* (DYT6)

DYT-*THAP1* (DYT6) is an early onset dystonia syndrome, with prominent oromandibular and laryngeal involvement at disease onset that typically spreads to the limbs, becoming more generalized (39, 40). The outcomes of GPi DBS in THAP1 dystonia appear poorer and more variable than idiopathic or DYT1 isolated dystonia with average improvements in BFMDS of around 35% and ranging from 16 to 72% with a tendency for limited or no improvement in speech or bulbar function (41–44). However, a few reports have described better outcomes including improvement in speech and swallowing (45, 46). There is some evidence that THAP1 dystonia may take longer to improve after GPi DBS than DYT1 or non-DYT1 dystonia but eventually responds to a similar degree albeit with more variable outcomes (47). A recent larger study of 14 THAP1 dystonia patients treated with GPi DBS with median follow up of 4 years found an average BFMDS improvement of 49% with limited improvements in speech noted, two non-responders and four patients with delayed worsening (48). Delayed worsening in THAP1 with improvement after lead repositioning has been reported in several patients (41, 43, 49). Concerns for effectiveness of GPi DBS in THAP1 dystonia have led to alternative DBS targets being explored. Mure et al. reported 80% improvement in BFMDS at 2 years following bilateral ventral lateral anterior (VLa) thalamic nucleus DBS in a single adult patient with THAP1 dystonia (50). The reasons for poorer and more variable DBS response in THAP1 dystonia are still not fully understood but may in part relate to prominent bulbar involvement which is a body region usually less responsive to DBS. Another possibility is genetic heterogeneity in DYT-THAP1 where many different pathogenic mutations have been described whereas DYT1 dystonia is usually due to a single common GAG deletion.

### DYT/PARK-*TAF1* (DYT3)

X-linked dystonia parkinsonism (XDP or “Lubag”) is associated with a SINE-VNTR-Alu retrotransposon insertion within an intron of the *TATA box-binding protein–associated factor 1* (*TAF1*) gene (51, 52). It affects individuals with maternal origin from the island of Panay, in the Philippines (53). In this disorder, affected individuals develop focal or segmental dystonia in adulthood; the dystonia rapidly becomes generalized over several years (51). About 5–10 years after the onset of disease, dystonia features become less prominent, and parkinsonian features predominate (51). The dystonia in XDP is severe and disabling, oral medications have inconsistent benefit and neuroablative therapy targeting the bilateral pallidus and/or thalamus has resulted in major adverse events; thus GPi-DBS has been used as a therapeutic option (51, 54–56).

Perhaps the most comprehensive study of GPi-DBS to date involved 16 males with XDP from the Philippines with predominant dystonia (51). There was an improvement in dystonia post-operatively as well as the Unified Parkinson's Disease Rating Scale Part III (UPDRS III). Additionally, T1-based basal ganglia volumetry showed that caudate atrophy was a predictor of a less beneficial outcome.

Improvements in both BFMDRS and UPDRS measures suggests that GPi-DBS can be beneficial for both dystonia and parkinsonian features in XDP (51). However, there may be a differential effect of bilateral GPi-DBS, with an marked, immediate and sustained improvement in dystonia but with a lesser benefit for parkinsonism (56–58).

### DYT-*TUBB4A* (DYT4)

Mutations in *TUBB4A* have been found to cause whispering dysphonia (DYT4)–spasmodic dysphonia combined with other focal or generalized dystonia, and a characteristic “hobby horse” gait. (59–61). Mutations in *TUBB4A* can also cause other neurological phenotypes such as hypomyelination with atrophy of the basal ganglia and cerebellum (H-ABC) syndrome (62) or hereditary spastic paraplegia (63).

There is a single case report documenting the response to DBS in TUBB4A-related dystonia. A 44 year old man with a p.Arg2Gly variant in *TUBB4A* was found to have an improvement in dystonia (55% reduction in BFMDRS) with a more prominent improvement in cervical and facial dystonia with bilateral GPi-DBS (64).

### DYT-*SGCE* (DYT11)

DYT11 is an autosomal dominantly inherited condition due to a heterozygous mutation in the *SGCE* gene, with paternal expression and reduced penetrance with maternal transmission (65). It results in early onset myoclonus dystonia usually presenting in childhood with upper body myoclonus and dystonia frequently with writer's cramp, cervical dystonia, and associated psychiatric problems including anxiety and obsessive compulsive disorder (66–68). The initial reports of beneficial effects of DBS in myoclonus dystonia were in patients without confirmation of *SCGE* mutation, where suppression of myoclonus without improvement in dystonia following staged bilateral ventralis intermediate nucleus (VIM) DBS was reported (69) and improvement in both myoclonus and dystonia following GPi DBS (70, 71). The first report of GPi DBS for genetically confirmed SGCE myoclonus dystonia was in an 8-year-old boy with marked improvement of both myoclonus and dystonia (72). Subsequently a number of studies reported good response and improvement in both dystonia and myoclonus in DYT11 dystonia following GPi DBS (73–78).

Kosutzka et al. reported long-term outcomes in 9 DYT-SCGE patients treated with bilateral GPi DBS who were followed up for minimum of 5 years. In this study motor improvement was marked with myoclonus improving by 94%, dystonia by 71%, 88% improvement in disability score and significantly improved function and social adjustment (79). A recent meta analysis of individual patient outcomes in 71 patients with myoclonus dystonia and GPi DBS found an average improvement in unified myoclonus rating scale of 79.5% and significant improvements in dystonia motor and disability scores with possible predictive factors for superior myoclonus outcome including shorter disease duration (80). Thalamic stimulation targeting the VIM nucleus appears to be an effective alternative to GPi DBS (73, 81), however pallidal stimulation is generally considered the preferred target (82). Patients with isolated myoclonus SCGE without dystonia also appear to benefit significantly from GPi DBS (83, 84).

While DBS can benefit the motor features of DYT-SGCE, it should be noted that there may not be a parallel improvement in psychiatric comorbidities (85).

### DYT-*ATP1A3* (DYT12)

Mutations in *ATP1A3* can causes a wide spectrum of clinical phenotypes including alternating hemiplegia of childhood type 2, cerebellar ataxia, areflexia, pes cavus, optic atrophy, and sensorineural hearing loss (CAPOS) syndrome, rapid-onset dystonia-parkinsonism (RDP, DYT12), as well as numerous “non-classical” phenotypes (86). There are case reports of DBS use in ATP1A3-related disorders, providing insights into the likely beneficial response.

One report of a 21 year old woman with presumed RDP who had bilateral GPi-DBS described no improvement in symptoms (87). A 12 year old boy with a novel, *de novo, ATP1A3* mutation had onset of dysphagia and dysarthria followed by severe generalized dystonia resulting in inability to walk (88). Following bilateral GPi-DBS he was temporarily able to walk with a temporal improvement in BFMDRS but only a marginal improvement in UPDRS. Two further reports suggested that GPi-DBS was ineffective in ATP1A3-related dystonia (89, 90). However, in a kindred with a novel *ATP1A3* variant causing generalized dystonia and paroxysmal dystonic attacks, bilateral GPi-DBS resulted in a remission of paroxysmal episodes and an improvement of interictal dystonia in one family member (91), suggesting that the response to DBS in ATP1A3-related dystonia is not uniformly poor but instead variable.

### DYT-*PRKRA* (DYT16)

Mutations in *PRKRA* cause young onset autosomal recessive dystonia parkinsonism (92). A response to GPi-DBS has been shown in a small number of cases of DYT16; a study of 2 patients showed an improvement in BFMDRS and walking times on gait analysis (93), and GPI-DBS produced a sustained improvement in cranial and limb dystonia over 10 years in one patient with a homozygous *PRKRA* mutation (94).

### DYT-*ANO3* (DYT24)

Mutations in *ANO3* have been associated with autosomal dominant, adult-onset craniocervical dystonia and dystonic tremor as well as myoclonic jerks (95).

To date, there have been at least 5 reported cases documenting a response to bilateral GPi-DBS in ANO3-related dystonia (96–100). For example, one patient with a *de novo* variant in *ANO3* (p.Val561Glu) and early onset, generalized dystonia was found to improve with bilateral GPi-DBS, resulting in a sustained benefit allowing her to walk with assistance (99). Another patient with an *ANO3* mutation (p.Glu510Lys) had a substantial improvement in dystonia and tremor but in this case myoclonus persisted (96). A further patient with ANO3-related dystonia had a successful response to GPi-DBS but continued to have episodes of dystonic storms (97). We conclude that DBS is likely to result in a successful but partial response in dystonia due to *ANO3* mutations, but large case series are needed.

### DYT-*GNAL* (DYT25)

Autosomal dominant mutations in the *GNAL* gene cause primary torsion dystonia, typically with a craniocervical onset, although progression to other sites and generalized dystonia can occur, with a phenotype resembling THAP1-associated dystonia (101–103).

There are several case reports or case series documenting a response to GPi-DBS in *GNAL* mutation carriers (104–108). One patient with a novel missense variant (p.Met97Val) in *GNAL* with an associated phenotype of late-onset cervical and truncal dystonia had excellent long term and continuous benefit from bilateral GPi-DBS at 5 years follow-up (104).

In a family with a novel *GNAL* mutation (p.Asp210Asn), the index patient with cervical dystonia and tremor derived a 67% improvement in BFMDRS following bilateral GPi-DBS (105). The index patient's younger sister also obtained a benefit from dystonia in terms of an improvement in cervical and laryngeal dystonia (105).

The largest case series so far documented the response of 3 unrelated individuals with isolated dystonia due to a mutation in *GNAL* (108). All patients improved with bilateral GPi-DBS, with a predominant improvement in cervical dystonia compared to other regions. However, a case report of an individual with a *GNAL* variant (p.Cys429Tyr) did report improvement in other anatomical regions following bilateral GPi-DBS (107).

In summary, according to the limited patients reported, GNAL-related dystonia is likely to result in a favorable though incomplete response to GPi-DBS.

### DYT-*KMT2B* (DYT28)

Heterozygous variants in the *KMT2B* gene are emerging as one of the commonest causes of early onset dystonia, with a caudocranial pattern evolving into generalized dystonia (109–111). There may be additional features, such as a characteristic facies, microcephaly, short stature, developmental delay, mild psychomotor impairment, and superimposed choreoathetosis or myoclonus (111, 112). Furthermore, MRI findings show characteristic changes including bilateral pallidal hypointensity that may serve as a clue to this disorder (110, 113).

While initially thought to be unresponsive to medical therapy (110), pharmacotherapy with trihexyphenidyl alone or in combination with clonazepam has been reported to reduce dystonia in some cases (114, 115). The reported response to bilateral GPi-DBS is more consistent, with improvements of motor function and gait, and restoration of walking in some patients (110).

A very recent study described the largest sample of KMT2B-related dystonia to date, including 18 individuals with medication-refractory dystonia (111). Significant improvement of motor function and disability [BFMDRS movement (BFMDRS-M) and BFMDRS disability (BFMDRS-D)] was evident at 6 months, 1 year and last follow-up (up to 22 years). Therefore, one of the characteristic features of dystonia due to *KMT2B* mutations is a response to DBS, which may be the preferred option in severely affected patients (116). The effect of DBS is seen in all anatomical regions, apart from perhaps a less beneficial effect upon laryngeal dystonia (111, 114).

### ADCY5-Related Dystonia

ADCY5-dyskinesia is inherited in an autosomal dominant manner and causes a spectrum of clinical features including chorea, athetosis, myoclonus, dystonia, and ballistic bouts, with an onset in infancy to late-adolescence (117, 118).

In the largest case series to date, 3 patients with *ADCY5* mutations has bilateral GPi-DBS (119). There was a subjective general improvement and a reduction in nocturnal episodic dyskinesias. However, on objective measures there was only a mild decrease in involuntary movements, and dystonia improved in 1 of 3 patients.

### GNAO1-Related Dystonia

*De novo* heterozygous mutations in *GNAO1* cause a spectrum of disorders including a neurodevelopmental delay, epileptic encephalopathy, and involuntary movements (120). There are numerous case reports and case series supporting DBS in GNAO1-associated movement disorders (120–124). Of note, emergency GPi-DBS in a severely ill patient produced a dramatic, life-saving response with almost complete remission of the hyperkinesia despite persistence of generalized dystonia (120). Furthermore, GPi-DBS was shown to be effective in two cases of status dystonicus due to *GNAO1* mutations.

### NBIA/DYT-*PANK2*

Biallelic mutations in *PANK2* cause neurodegeneration with brain iron accumulation-1 also known as pantothenate kinase-associated neurodegeneration (PKAN), and previously known as Hallervorden-Spatz disease (125). The phenotype may be classic (early onset dystonia, choreoathetosis, dysarthria and rigidity) or atypical (late onset or slowly progressive forms) (19, 126). The MRI findings including iron deposition in the basal ganglia which may result in the characteristic “eye of the tiger sign” (126).

There are several case reports of dystonia due to PKAN treated with GPi-DBS, with most documenting a good response (127–131), although with one instance of no benefit (132). It is notable that genetic testing was not always performed in these cases, making it difficult to be sure of the genotype specific outcome.

A study of 6 individuals with *PANK2* mutation-positive PKAN showed a major and sustained improvement in painful spasms, dystonia, and ambulation (19). A further study from Korea demonstrated a benefit in 2 patients with atypical PKAN but a variable benefit in 2 individuals with typical PKAN (133).

The largest study of NBIA to date was of 16 centers contributing 23 individuals who underwent GPi-DBS (20). Fifteen of those had genetic testing, and 14 were found to have *PANK2* mutations. They reported an improvement in dystonia severity, disability, and quality of life. However, the improvement was not as great as those with primary generalized dystonia or other secondary dystonias. Additionally, patients with more severe dystonia appeared to derive greater benefit.

## Other Genetic Causes of Dystonia

There are rarer forms of monogenic dystonia in whom a response to DBS has been reported but the evidence is limited so far. STN-DBS was found to be efficacious in two patients with *GCH1* variants for parkinsonism and motor fluctuations following long-term treatment with levodopa (134). A case report of GNB1-related myoclonus dystonia showed an initial marked response to GPi-DBS (135). KCTD17-related myoclonus dystonia is known to have an excellent response to GPi-DBS (136), including an improvement in orolingual dyskinesia and speech (137). In a patient with deafness dystonia syndrome due to an *ACTB* mutation (p.Arg183Trp), GPi-DBS was also found to be effective (138).

A further case study reported 2 patients with double mutations in DYT1 and DYT11, who a successful response to bilateral VIM DBS (followed by GPi-DBS in one patient) (139).

Heterozygous loss of function mutations in *VPS16* have recently been found to cause dystonia with prominent craniocervical and upper limb involvement (140). It is notable that some patients experience an improvement from DBS (140), although further data is required.

GM1 gangliosidosis is one several neurometabolic causes of dystonia (141). A patient with GM1 type 3 gangliosidosis was reported was reported to have a significant functional benefit but no change to disease progression with bilateral GPi-DBS (142).

## Systematic Reviews

A very recent systematic review and meta-analysis of GPi-DBS for monogenic dystonia found robust support for DYT1, modest support for DYT6 and PANK2-related dystonia, and promising results for SGCE, DYT3, ACTB and GNAO1-related dystonia, supporting the concept of a differential outcomes for the individual monogenic forms (143). An early age at onset (DYT1 and SGCE) was associated with better outcomes. Moreover, a shorter duration prior to GPi-DBS (DYT1 and DYT3) was associated with a better outcome, suggesting that perhaps earlier intervention would be beneficial in these individuals (143).

A recent systematic review demonstrated an improvement in physical quality of life, but the improvement in mental quality of life was less robust (144).

## Conclusion

**Figure 1:**
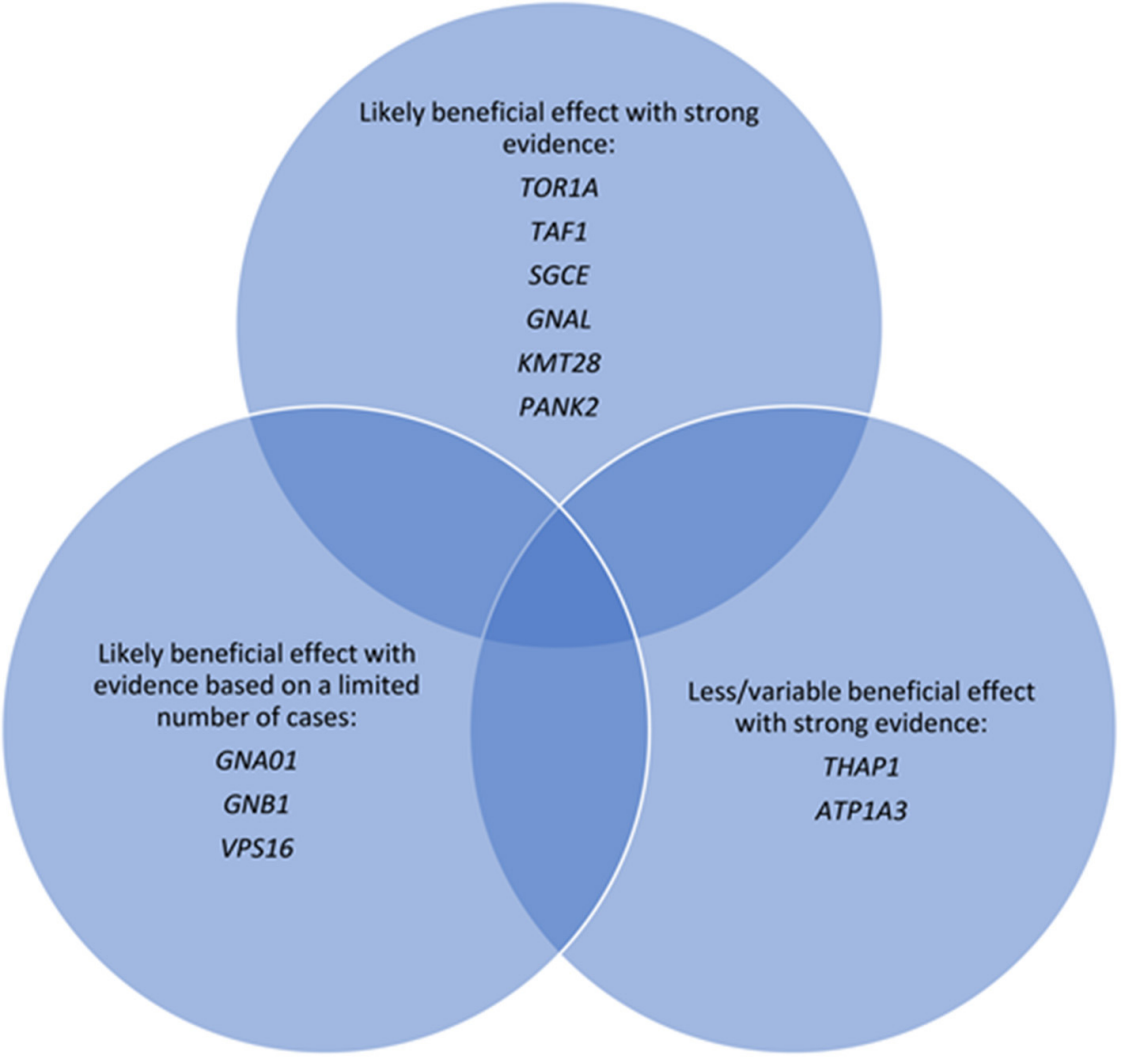
Major monogenic forms of dystonia categorized according to evidence of response to deep brain stimulation.

Pallidal DBS is effective across a range of monogenic forms of DBS, with a suggestion of a gene-specific differential effect (Figure 1). However, the evidence is limited by small cohort sizes or case reports, particularly for the rarer subtypes. Within these limitations, patterns of response between different monogenic dystonias may assist in patient selection for DBS and determining treatment prognosis, particularly if additional factors such as body distribution, disease duration or the presence of orthopedic deformity are included in the overall assessment. Patients should still be warned of the small risks of treatment failure or secondary worsening, even those with monogenic dystonias considered highly responsive to DBS. In this context the genetic diagnosis forms one part of the decision-making algorithm and perhaps with the exception of DYT-*ATP1A3*, finding a particular gene should not discourage consideration of GPi DBS if alleviation of a disabling dystonic syndrome is clinically imperative. Systematic reviews can be helpful as described (143), and international registries may address this issue further (145), particularly if the collection of clinical data is uniform, allowing for comparison between centers.

We highlight the importance of identifying a genetic diagnosis in dystonia, which, aside from multiple benefits including assistance with genetic counseling and family planning, may help guide expectations as to the likely outcome of DBS, for both patients and clinicians. This supports the concept of routine genetic testing of patients with dystonia prior to DBS (146). In this case, the options would be targeted gene panel sequencing, whole exome sequencing, or whole genome sequencing (96, 113)–the most cost effective approach is yet to be determined. An additional advantage of a genetic diagnosis in dystonia patients being considered for DBS is that it effectively excludes an unrecognized acquired combined (secondary) etiology where a poorer DBS outcome would be predicted.

It is uncertain whether GPi-DBS should be reserved for severe, medication-resistant cases, or whether it should be instituted at a much earlier point. What is well-accepted is that DBS should be considered in medically refractory dystonia before orthopedic deformity has developed. DBS responses may be variable and depend on other factors such as age (146). Furthermore, DBS may have a differential effect for certain phenotypic manifestations (e.g., improvement in dystonia greater than parkinsonism for DYT/PARK-*TAF1* or limited improvement in speech and swallowing in DYT-*THAP1*).

While genetic testing may have a role with guiding expectations in GPi-DBS for genetic forms of dystonia, ultimately to decision to proceed should be based on the clinical phenotype.

## Author Contributions

ST and KK contributed to the first draft and revision of the manuscript. Both authors contributed to the article and approved the submitted version.

## Conflict of Interest

The authors declare that the research was conducted in the absence of any commercial or financial relationships that could be construed as a potential conflict of interest.
