# Supplementary material for: Pallidal Deep Brain Stimulation for Monogenic Dystonia: The Effect of Gene on Outcome
Source: Front Neurol. 2021 Jan 8;11:630391. doi: 10.3389/fneur.2020.630391 (PMC7820073; doi:10.3389/fneur.2020.630391)
Supplement: Supplementary file 1 [file Table_1.docx]

Supplementary Material

**Supplementary Table 1. Major studies of DBS in monogenic dystonia***

| Gene | Traditional nomenclature | MDSGene nomenclature | Inclusion criteria | Number of patients | DBS target | Study duration | Outcome measures | Result | Reference |
| --- | --- | --- | --- | --- | --- | --- | --- | --- | --- |
| *TOR1A* | DYT1 | DYT-*TOR1A* | DYT1 | 26 | Bilateral GPi | Up to 10 years follow up | BFMDRS-M/D | Marked sustained improvement | (Cif et al., 2010) |
|  |  |  | DYT1 | 47 | Bilateral GPi | Average follow up 46 months | BFMDRS-M/D | 80% improvement in motor score | (Panov et al., 2013) |
| *THAP1* | DYT6 | DYT-*THAP1* | Isolated dystonia including DYT1, non-DYT1 and DYT6 | DYT6 n=8 | Bilateral GPi | Up to 92 months | BFMDRS-M | 32% “early” improvement (1-16 months)  42% “late” improvement (22-92 months) | (Bruggemann et al., 2015) |
|  |  |  | DYT6 | 14 | Bilateral GPi | Median 4 years, up to 10 months follow up | BFMDRS-M | 49% average improvement in motor score | (Danielsson et al., 2019) |
| TAF1 | DYT3 | DYT/PARK-*TAF1* | XDP patients | 3 | Bilateral GPi | Average follow-up 45.7 months | BFMDRS-M, UPDRS-III | Immediate and sustained improvement in dystonia (mean percentage improvement in BFMDRS-M was 63.5%), Parkinsonism was less responsive (mean improvement in UPDRS-III of 39.5%) | (Kilbane et al., 2018) |
|  |  |  | XDP patients | 11 | Bilateral GPi | Up to 84 months | BFMDRS-M, UPDRS-III | Effective in the first 12 months for dystonia, variable response in controlling parkinsonism, improvement in clinical and functional stage | (Abejero et al., 2019) |
|  |  |  | Men with XDP from the Phillipines with predominant dystonia | 16 | Bilateral GPi | Up to 46 months | BFMDRS-M, UPDRS-III, T1-based basal ganglia volumetry | Improvement in masked video ratings of dystonia and UPDRS-III, caudate atrophy may predict a poorer outcome | (Bruggemann et al., 2019) |
| *SGCE* | DYT11 | DYT-*SGCE* | *SGCE* mutation-positive myoclonus dystonia | 5 | Bilateral GPi | Assessments at 6 to 9 months and 15 to 18 months post-operatively | BFMDRS, UMRS | Improvement in myoclonus scores, dystonia scores and disability | (Azoulay-Zyss et al., 2011) |
|  |  |  | *SGCE* mutation-positive myoclonus dystonia | 3 | Bilateral GPi | 18-30 months | BFMDRS, UMRS | Improves BFMDRS, UMRS | (Fernandez-Pajarin et al., 2016) |
|  |  |  | Myoclonus dystonia | Two positive and three negative *SGCE* mutation patients | Vim (bilateral in 4 patients, unilateral in 1) | Average follow-up 50 months | BFMDRS, UMRS | Greater improvement in myoclonus compared to BFMDRS motor score and disability score | (Zhang et al., 2019) |
|  |  |  | *SGCE* mutation-positive myoclonus dystonia | 9 | Bilateral GPi | 5 years | BFMDRS, UMRS, Abnormal Involuntary Movement Scale, standardized video-protocols, evaluation of social adjustment, cognition, and mood | Improvement in the composite myoclonus score, dystonia severity improved, motor disability improved, abnormal involuntary movement score improved, no major postoperative complications | (Kosutzka et al., 2019) |
| *PRKRA* | DYT16 | DYT-*PRKRA* | DYT16 with isolate generalised dystonia | 2 | Bilateral GPi | 6 months | BFMDRS-M/D, Patient’s Global Impression of Change Scale, gait analysis | Improvement in BFMDRS-M/D and walking times | (Casagrande et al., 2019) |
| *GNAL* | DYT25 | DYT-*GNAL* | Family with a novel *GNAL* mutation | 2 | Bilateral GPi | Up to 4 years | BFMDRS | Improvement in dystonia, improvement in BFMDRS in the index patient | (Carecchio et al., 2016) |
|  |  |  | *GNAL* mutation, cases were ascertained post-DBS | 3 | Bilateral GPi | Up to 2 years | Clinical assessment, TWSTRS | Successful (but incomplete) response, improvement in TWSTRS | (Sarva et al., 2019) |
| *KMT2B* | DYT28 | DYT-*KMT2B* | Microdeletion or *KMT2B* mutation positive | 10 | Bilateral GPi | Up to 2 years | Clinical assessment | All had a clinical response | (Meyer et al., 2017) |
|  |  |  | *KMT2B* mutations | 3 | Bilateral GPi | 1 month | Clinical assessment and BFMDRS | Improvement in BFMDRS, Excellent or partial response | (Kawarai et al., 2018) |
|  |  |  | *KMT2B* mutations | 2 | Bilateral GPi | Up to 10 years | Clinical assessment and BFMDRS-M | Improvement in motor performance, ambulation, BFMDRS-M | (Dafsari et al., 2019) |
|  |  |  | *KMT2B* mutations | 8 | Bilateral GPi | Median postoperative follow-up was 12 years (range, 8–17) | BFMDRS-M | Median decrease of BFMDRS-M of 38.5% in the long term | (Carecchio et al., 2019) |
|  |  |  | *KMT2B* mutation carrier | 9 | 8 targeted the GPi, 1 targeted the STN | Mean follow-up time was 4.9 (range 1.3–16) months | Clinical evaluation, video, BFMDRS-M/D | Dramatic clinical improvement, improvement in BFMDRS-M/D | (Li et al., 2020) |
|  |  |  | *KMT2B* mutation carrier# | 18 | Bilateral GPi | 0.25 to 22 years | BFMDRS-M/D | Significant improvement at 6 months, 1 year and last follow-up | (Cif, 2020) |
| *ADCY5* | Familial dyskinesia with facial myokymia | CHOR/DYT-*ADCY5* | ADCY5-related hyperkinetic movement disorder | 3 | Bilateral GPi | Up to 13 year follow-up | Video, AIMS and BFMDRS | Subjective general improvement, long term reduction in nocturnal dyskinesias, mild decrease in involuntary movements and improvement in dystonia in 1 patient | (de Almeida Marcelino et al., 2020) |
| *GNAO1* | Developmental and epileptic encephalopathy 17, neurodevelopmental disorder with involuntary movements | - | Status dystonicus due to a *GNAO1* mutation | 2 | Bilateral GPi | 4.5 and 8 years | Clinical assessment | Return to baseline | (Benato et al., 2019) |
| *PANK2* | NBIA1 | NBIA/DYT-*PANK2* | Generalised dystonia with PKAN and *PANK2* mutation-positive | 6 | Bilateral GPi | Range 6-42 months | BFMDRS-M/D | Improvement in BFMDRS-M/D, painful muscle spasms and ambulation | (Castelnau et al., 2005) |
|  |  |  | NBIA | 23 individuals with NBIA, 14/15 in whom genetic testing revealing *PANK2* mutations | Bilateral GPi | Assessments 2–6 and 9–15 months postoperatively | BFMDRS-M/D, Barry Albright Dystonia Scale, quality of life | Improvement in dystonia severity, disability, and quality of life | (Timmermann et al., 2010) |
|  |  |  | PKAN due to *PANK2* mutations | 4 | Bilateral GPi | Up to 12 months | BFMDRS | Favorable outcome in two patients with atypical PKAN, variable outcome in 2 patients with typical PKAN | (Lim et al., 2012) |

*Studies including more than one unrelated subject were included.

#Study includes previously reported cases.

We included updated MDSGene nomenclature for genetic movement disorders (Marras et al., 2016).

AIMS - Abnormal Involuntary Movement Scale; BFMDRS - Burke‐Fahn‐Marsden Dystonia Rating Scale; BFMDRS –M – BFMDRS for movement; BFMDRS–M/D – BFMDRS for movement and disability; DBS – deep brain stimulation; GPi - globus pallidus pars interna; NBIA - neurodegeneration with brain iron accumulation; PKAN - pantothenate kinase-associated neurodegeneration; UMRS - Unified Myoclonus Rating Scale; UPDRS-III - United Parkinson’s disease rating Scale Part III, STN- subthalamic nucleus; TWSTRS - Toronto Western Spasmodic Torticollis Rating Scale; XDP - X-linked dystonia parkinsonism

REFERENCES

Abejero, J.E.E., Jamora, R.D.G., Vesagas, T.S., Teleg, R.A., Rosales, R.L., Anlacan, J.P., Velasquez, M.S., and Aguilar, J.A. (2019). Long-term outcomes of pallidal deep brain stimulation in X-linked dystonia parkinsonism (XDP): Up to 84 months follow-up and review of literature. *Parkinsonism Relat Disord* 60**,** 81-86. doi:10.1016/j.parkreldis.2018.09.022

Azoulay-Zyss, J., Roze, E., Welter, M.L., Navarro, S., Yelnik, J., Clot, F., Bardinet, E., Karachi, C., Dormont, D., Galanaud, D., Pidoux, B., Cornu, P., Vidailhet, M., and Grabli, D. (2011). Bilateral deep brain stimulation of the pallidum for myoclonus-dystonia due to epsilon-sarcoglycan mutations: a pilot study. *Arch Neurol* 68**,** 94-98. doi:10.1001/archneurol.2010.338

Benato, A., Carecchio, M., Burlina, A., Paoloni, F., Sartori, S., Nosadini, M., D'avella, D., Landi, A., and Antonini, A. (2019). Long-term effect of subthalamic and pallidal deep brain stimulation for status dystonicus in children with methylmalonic acidemia and GNAO1 mutation. *J Neural Transm (Vienna)* 126**,** 739-757. doi:10.1007/s00702-019-02010-2

Bruggemann, N., Domingo, A., Rasche, D., Moll, C.K.E., Rosales, R.L., Jamora, R.D.G., Hanssen, H., Munchau, A., Graf, J., Weissbach, A., Tadic, V., Diesta, C.C., Volkmann, J., Kuhn, A., Munte, T.F., Tronnier, V., and Klein, C. (2019). Association of Pallidal Neurostimulation and Outcome Predictors With X-linked Dystonia Parkinsonism. *JAMA Neurol* 76**,** 211-216. doi:10.1001/jamaneurol.2018.3777

Bruggemann, N., Kuhn, A., Schneider, S.A., Kamm, C., Wolters, A., Krause, P., Moro, E., Steigerwald, F., Wittstock, M., Tronnier, V., Lozano, A.M., Hamani, C., Poon, Y.Y., Zittel, S., Wachter, T., Deuschl, G., Kruger, R., Kupsch, A., Munchau, A., Lohmann, K., Volkmann, J., and Klein, C. (2015). Short- and long-term outcome of chronic pallidal neurostimulation in monogenic isolated dystonia. *Neurology* 84**,** 895-903. doi:10.1212/WNL.0000000000001312

Carecchio, M., Invernizzi, F., Gonzalez-Latapi, P., Panteghini, C., Zorzi, G., Romito, L., Leuzzi, V., Galosi, S., Reale, C., Zibordi, F., Joseph, A.P., Topf, M., Piano, C., Bentivoglio, A.R., Girotti, F., Morana, P., Morana, B., Kurian, M.A., Garavaglia, B., Mencacci, N.E., Lubbe, S.J., and Nardocci, N. (2019). Frequency and phenotypic spectrum of KMT2B dystonia in childhood: A single-center cohort study. *Mov Disord*. doi:10.1002/mds.27771

Carecchio, M., Panteghini, C., Reale, C., Barzaghi, C., Monti, V., Romito, L., Sasanelli, F., and Garavaglia, B. (2016). Novel GNAL mutation with intra-familial clinical heterogeneity: Expanding the phenotype. *Parkinsonism Relat Disord* 23**,** 66-71. doi:10.1016/j.parkreldis.2015.12.012

Casagrande, S.C.B., Listik, C., Coelho, D.B., Limongi, J.C.P., Teixeira, L.A., Teixeira, M.J., Barbosa, E.R., and Cury, R.G. (2019). Deep Brain Stimulation in Patients with Isolated Generalized Dystonia Caused by PRKRA Mutation. *Mov Disord Clin Pract* 6**,** 616-618. doi:10.1002/mdc3.12811

Castelnau, P., Cif, L., Valente, E.M., Vayssiere, N., Hemm, S., Gannau, A., Digiorgio, A., and Coubes, P. (2005). Pallidal stimulation improves pantothenate kinase-associated neurodegeneration. *Ann Neurol* 57**,** 738-741. doi:10.1002/ana.20457

Cif, L., Demailly, D., Lin, J.P., Barwick, K.E., Sa, M., Abela, L., Malhotra, S., Chong, W.K., Steel, D., Sanchis-Juan, A., Ngoh, A., Trump, N., Meyer, E., Vasques, X., Rankin, J., Allain, M.W., Applegate, C.D., Isfahani, S.A., Baleine, J., Balint, B., Bassetti, J.A., Baple, E.L., Bhatia, K.P., Blanchet, C., Burglen, L., Cambonie, G., Seng, E.C., Bastaraud, S.C., Cyprien, F., Coubes, C., D’hardemare, V., Deciphering Developmental Disorders Study, Doja, A., Dorison, N., Doummar, D., Dy-Hollins, M.E., Farrelly, E., Fitzpatrick, D.R., Fearon, C., Fieg, E.L, Fogel, B.L., Forman, E.B., Fox, R., Genomics England Research Consortium, Gahl, W.A., Galosi, S., Gonzalez, V., Graves, T.D., Gregory, A., Hallett, M., Hasegawa, H., Hayflick, S.J., Hamosh, A., Hully, M., Jansen, S., Jeong, S.Y., Krier, J.B., Krystal, S., Kumar, K.R., Laurencin, C., Lee, H., Lesca, G., François, L.L., Lynch, T., Mahant, N., Martinez-Agosto, J.A., Milesi, C., Mills, K.A., Mondain, M., Morales-Briceno, H., Nihr Bioresource, Ostergaard, J.R., Pal, S., Pallais, J.C., Pavillard, F., Perrigault, P.F., Petersen, A.K., Polo, G., Poulen, G., Rinne, T., Roujeau, T., Rogers, C., Roubertie, A., Sahagian, M., Selim, L., Selway, R., Sharma, N., Signer, R., Soldatos, A.G., Stevenson, D.A., Stewart, F., Tchan, M., Undiagnosed Diseases Network, Verma, I.C., De Vries, B.B.A., Wilson, J.L., Wong, D.A., Zaitoun, R., Zhen, D., Znaczko, A., Dale, R.C., De Gusmão, C.M., Friedman, J., Fung, V.S.C., King, M.D., Mohammad, S.S., Rohena, L., Waugh, J.L., Toro, C., Raymond, F.L., Topf, M., Coubes, P., Gorman, K.M. And Kurian, M.A. (2020). KMT2B-related disorders: Expansion of the phenotypic spectrum and long-term efficacy of deep brain stimulation. *Brain* In press.

Cif, L., Vasques, X., Gonzalez, V., Ravel, P., Biolsi, B., Collod-Beroud, G., Tuffery-Giraud, S., Elfertit, H., Claustres, M., and Coubes, P. (2010). Long-term follow-up of DYT1 dystonia patients treated by deep brain stimulation: an open-label study. *Mov Disord* 25**,** 289-299. doi:10.1002/mds.22802

Dafsari, H.S., Sprute, R., Wunderlich, G., Daimaguler, H.S., Karaca, E., Contreras, A., Becker, K., Schulze-Rhonhof, M., Kiening, K., Karakulak, T., Kloss, M., Horn, A., Pauls, A., Nurnberg, P., Altmuller, J., Thiele, H., Assmann, B., Koy, A., and Cirak, S. (2019). Novel mutations in KMT2B offer pathophysiological insights into childhood-onset progressive dystonia. *J Hum Genet* 64**,** 803-813. doi:10.1038/s10038-019-0625-1

Danielsson, A., Carecchio, M., Cif, L., Koy, A., Lin, J.P., Solders, G., Romito, L., Lohmann, K., Garavaglia, B., Reale, C., Zorzi, G., Nardocci, N., Coubes, P., Gonzalez, V., Roubertie, A., Collod-Beroud, G., Lind, G., and Tedroff, K. (2019). Pallidal Deep Brain Stimulation in DYT6 Dystonia: Clinical Outcome and Predictive Factors for Motor Improvement. *J Clin Med* 8. doi:10.3390/jcm8122163

De Almeida Marcelino, A.L., Mainka, T., Krause, P., Poewe, W., Ganos, C., and Kuhn, A.A. (2020). Deep brain stimulation reduces (nocturnal) dyskinetic exacerbations in patients with ADCY5 mutation: a case series. *J Neurol*. doi:10.1007/s00415-020-09871-8

Fernandez-Pajarin, G., Sesar, A., Relova, J.L., Ares, B., Jimenez-Martin, I., Blanco-Arias, P., Gelabert-Gonzalez, M., and Castro, A. (2016). Bilateral pallidal deep brain stimulation in myoclonus-dystonia: our experience in three cases and their follow-up. *Acta Neurochir (Wien)* 158**,** 2023-2028. doi:10.1007/s00701-016-2904-3

Kawarai, T., Miyamoto, R., Nakagawa, E., Koichihara, R., Sakamoto, T., Mure, H., Morigaki, R., Koizumi, H., Oki, R., Montecchiani, C., Caltagirone, C., Orlacchio, A., Hattori, A., Mashimo, H., Izumi, Y., Mezaki, T., Kumada, S., Taniguchi, M., Yokochi, F., Saitoh, S., Goto, S., and Kaji, R. (2018). Phenotype variability and allelic heterogeneity in KMT2B-Associated disease. *Parkinsonism Relat Disord* 52**,** 55-61. doi:10.1016/j.parkreldis.2018.03.022

Kilbane, C., Witt, J., Galifianakis, N.B., Glass, G.A., Volz, M., Heath, S., Starr, P.A., and Ostrem, J.L. (2018). Long-Term Outcomes of Bilateral Pallidal Deep Brain Stimulation for X-Linked Dystonia and Parkinsonism. *Stereotact Funct Neurosurg* 96**,** 320-326. doi:10.1159/000492823

Kosutzka, Z., Tisch, S., Bonnet, C., Ruiz, M., Hainque, E., Welter, M.L., Viallet, F., Karachi, C., Navarro, S., Jahanshahi, M., Rivaud-Pechoux, S., Grabli, D., Roze, E., and Vidailhet, M. (2019). Long-term GPi-DBS improves motor features in myoclonus-dystonia and enhances social adjustment. *Mov Disord* 34**,** 87-94. doi:10.1002/mds.27474

Li, X.Y., Dai, L.F., Wan, X.H., Guo, Y., Dai, Y., Li, S.L., Fang, F., Wang, X.H., Zhang, W.H., Liu, T.H., Xie, Z.H., Fang, T., Wang, L., and Ding, C.H. (2020). Clinical phenotypes, genotypes and treatment in Chinese dystonia patients with KMT2B variants. *Parkinsonism Relat Disord* 77**,** 76-82. doi:10.1016/j.parkreldis.2020.06.002

Lim, B.C., Ki, C.S., Cho, A., Hwang, H., Kim, K.J., Hwang, Y.S., Kim, Y.E., Yun, J.Y., Jeon, B.S., Lim, Y.H., Paek, S.H., and Chae, J.H. (2012). Pantothenate kinase-associated neurodegeneration in Korea: recurrent R440P mutation in PANK2 and outcome of deep brain stimulation. *Eur J Neurol* 19**,** 556-561. doi:10.1111/j.1468-1331.2011.03589.x

Marras, C., Lang, A., Van De Warrenburg, B.P., Sue, C.M., Tabrizi, S.J., Bertram, L., Mercimek-Mahmutoglu, S., Ebrahimi-Fakhari, D., Warner, T.T., Durr, A., Assmann, B., Lohmann, K., Kostic, V., and Klein, C. (2016). Nomenclature of genetic movement disorders: Recommendations of the international Parkinson and movement disorder society task force. *Mov Disord* 31**,** 436-457. doi:10.1002/mds.26527

Meyer, E., Carss, K.J., Rankin, J., Nichols, J.M., Grozeva, D., Joseph, A.P., Mencacci, N.E., Papandreou, A., Ng, J., Barral, S., Ngoh, A., Ben-Pazi, H., Willemsen, M.A., Arkadir, D., Barnicoat, A., Bergman, H., Bhate, S., Boys, A., Darin, N., Foulds, N., Gutowski, N., Hills, A., Houlden, H., Hurst, J.A., Israel, Z., Kaminska, M., Limousin, P., Lumsden, D., Mckee, S., Misra, S., Mohammed, S.S., Nakou, V., Nicolai, J., Nilsson, M., Pall, H., Peall, K.J., Peters, G.B., Prabhakar, P., Reuter, M.S., Rump, P., Segel, R., Sinnema, M., Smith, M., Turnpenny, P., White, S.M., Wieczorek, D., Wiethoff, S., Wilson, B.T., Winter, G., Wragg, C., Pope, S., Heales, S.J., Morrogh, D., Consortium, U.K., Deciphering Developmental Disorders, S., Consortium, N.B.R.D., Pittman, A., Carr, L.J., Perez-Duenas, B., Lin, J.P., Reis, A., Gahl, W.A., Toro, C., Bhatia, K.P., Wood, N.W., Kamsteeg, E.J., Chong, W.K., Gissen, P., Topf, M., Dale, R.C., Chubb, J.R., Raymond, F.L., and Kurian, M.A. (2017). Mutations in the histone methyltransferase gene KMT2B cause complex early-onset dystonia. *Nat Genet* 49**,** 223-237. doi:10.1038/ng.3740

Panov, F., Gologorsky, Y., Connors, G., Tagliati, M., Miravite, J., and Alterman, R.L. (2013). Deep brain stimulation in DYT1 dystonia: a 10-year experience. *Neurosurgery* 73**,** 86-93; discussion 93. doi:10.1227/01.neu.0000429841.84083.c8

Sarva, H., Trosch, R., Kiss, Z.H.T., Furtado, S., Luciano, M.S., Glickman, A., Raymond, D., Ozelius, L.J., Bressman, S.B., and Saunders-Pullman, R. (2019). Deep Brain Stimulation in Isolated Dystonia With a GNAL Mutation. *Mov Disord* 34**,** 301-303. doi:10.1002/mds.27585

Timmermann, L., Pauls, K.A., Wieland, K., Jech, R., Kurlemann, G., Sharma, N., Gill, S.S., Haenggeli, C.A., Hayflick, S.J., Hogarth, P., Leenders, K.L., Limousin, P., Malanga, C.J., Moro, E., Ostrem, J.L., Revilla, F.J., Santens, P., Schnitzler, A., Tisch, S., Valldeoriola, F., Vesper, J., Volkmann, J., Woitalla, D., and Peker, S. (2010). Dystonia in neurodegeneration with brain iron accumulation: outcome of bilateral pallidal stimulation. *Brain* 133**,** 701-712. doi:10.1093/brain/awq022

Zhang, Y.Q., Wang, J.W., Wang, Y.P., Zhang, X.H., and Li, J.P. (2019). Thalamus Stimulation for Myoclonus Dystonia Syndrome: Five Cases and Long-Term Follow-up. *World Neurosurg* 122**,** e933-e939. doi:10.1016/j.wneu.2018.10.177
